# Roles of AFP, AFP-L3, DCP and GP73 in Diagnosis of Hepatocellular Carcinoma and Prediction of Recurrence in Patients

**DOI:** 10.7150/jca.125861

**Published:** 2026-01-01

**Authors:** Yuan Liao, Ziying Mo, Cailing Ye, Yaqiong Chen, Huimin Dong, Bo Hu

**Affiliations:** Department of Laboratory Medicine, The Third Affiliated Hospital of Sun Yat-sen University, Guangzhou 510630, China.

**Keywords:** hepatocellular carcinoma (HCC), alpha-fetoprotein (AFP), Des-gamma carboxy-prothrombin (DCP), lectin-bound AFP (AFP-L3), Golgi protein-73 (GP73)

## Abstract

**Background:** Alpha-fetoprotein (AFP), Des-gamma carboxy-prothrombin (DCP), lectin-bound AFP (AFP-L3) and Golgi protein-73 (GP73) have been used or proposed as surveillance tests for hepatocellular carcinoma (HCC). The aims of this study were to determine the performance of AFP, DCP, AFP-L3, GP73 and their combination in the diagnosis and prognosis of HCC.

**Methods:** A total of 578 patients were enrolled, including 303 HCC patients, 104 patients with liver cirrhosis, 101 patients with chronic hepatitis and 70 healthy volunteers. The serum levels of AFP, DCP, AFP-L3 and GP73 were quantified before treatment, 7 days and 30 days after treatment.

**Results:** AFP had the best area under the curve (AUC = 0.850), followed by DCP (0.775) and AFP-L3 (0.763), for the prediction of HCC, whereas GP73 had low diagnostic value (0.549). The combination of AFP, DCP and AFP-L3 significantly improved diagnostic performance (AUC = 0.895). The level of AFP 30 days after treatment had the best predictive value for HCC recurrence (AUC = 0.779). Higher recurrence rates were associated with an increasing number of elevated tumor markers measured both before and 30 days after treatment. Furthermore, patients whose marker status remained positive 30 days after treatment had a higher recurrence rate than patients whose marker status changed to negative.

**Conclusions:** AFP was more effective than DCP and AFP-L3 for the diagnosis and prognosis of HCC, and the combination of AFP, AFP-L3 and DCP enhanced the diagnostic performance. The dynamic changes in biomarker positive status after treatment and the number of positive biomarkers play important roles in predicting HCC recurrence.

## Introduction

Hepatocellular carcinoma (HCC) is among the most common tumor types, and its incidence and mortality are increasing worldwide 1, 2. Early diagnosis of HCC is very important since curative therapies are only available for early-stage HCC 3. Hepatitis B virus (HBV) is the leading cause of HCC worldwide, particularly in Asia and Africa 4. Therefore, surveillance of nonmalignant chronic liver diseases associated with a high risk of HCC is key to improve the poor prognosis of HCC.

Ultrasound (US) is the main recommended tool in HCC surveillance 5. However, it presents rather limited sensitivity in detecting early HCC and offers heterogeneous results according to the expertise of the operator and the quality of the equipment 6. Tumor biomarkers for early HCC diagnosis and determination of prognosis are still lacking and may have an increased clinical role in the near future. Presently, several biomarkers have been used clinically or are under investigation for the early diagnosis of HCC, including alpha-fetoprotein (AFP), des-gamma-carboxy prothrombin (DCP) and Lens culinaris agglutinin-reactive fraction of AFP (AFP-L3) and Golgi protein-73 (GP73). AFP is the most widely used biomarker in HCC surveillance. However, AFP is positive in only 60%-80% of HCCs, and AFP can be elevated in other benign or malignant conditions, such as chronic hepatitis, cirrhosis, intrahepatic cholangiocarcinoma and embryogenic tumors, leading to an unreliable role of AFP in surveillance 7. AFP-L3, as a fucosylated variant of AFP, is considered a more specific biomarker for HCC than AFP since it is produced exclusively by HCC cells 8. Although AFP-L3 displayed an extremely high specificity of 92-97% in multicenter studies, its low sensitivity of 28-37% limits its potential as an HCC biomarker alone 9, 10. DCP, known as protein induced by vitamin K absence/antagonist-II (PIVKA-II), has been described as a useful tool for HCC surveillance since it is independent of AFP secretion. However, its efficacy as a screening tool is still controversial and requires further investigation, particularly in combination with AFP 11, 12. Golgi protein-73 (GP73), a resident Golgi glycoprotein, is upregulated in serum samples from patients with liver diseases, especially those with HCC, and is expected to be a new serum marker for the diagnosis of HCC 13. However, this hypothesis needs to be proven in large cohorts. In addition to their use as diagnostic tools for surveillance, tumor biomarkers play important roles as predictors of patient outcome. Many studies have shown that AFP, AFP-L3, DCP and GP73 are associated with HCC prognosis 13-15. However, most of these studies focused on pretreatment levels while ignoring the potential predictive value of post-treatment levels and their dynamic changes in the context of HCC treatment.

In the present study, we aimed to compare the diagnostic performance of the four biomarkers for detecting HCC and construct multimarker prediction algorithms to distinguish HCC from nonmalignant chronic liver diseases. Moreover, we measured the levels of the four tumor biomarkers both before and after treatment and analyzed their ability to predict tumor recurrence after treatment.

## Materials and Methods

### Patients

This study protocol was approved by the Ethics Committee of the Third Affiliated Hospital of Sun Yat-sen University as stipulated by the Declaration of Helsinki, and written informed consent was obtained from all subjects.

From April 2018 to December 2019, a total of 578 patients were enrolled in the Third Affiliated Hospital of Sun Yat-sen University, including 303 HCC patients, 104 patients with liver cirrhosis (LC), 101 patients with chronic hepatitis (CH) and 70 healthy controls (HC). The diagnosis of HCC was based on the diagnostic criteria for HCC used by the European Association for the Study of the Liver 16. The inclusion criteria were as follows: 1) HCC with no previous treatment; 2) Eastern Cooperative Oncology Group Performance Status of 0-1; and 3) Child‒Pugh classification of A or B. The diagnosis of cirrhosis was based on liver histology or clinical, laboratory, and imaging evidence of hepatic decompensation or portal hypertension. Chronic hepatitis was defined as an inflammatory disease of the liver without improvement for at least six months. The inflammatory reaction is demonstrated by persistently abnormal liver function tests and by histological changes. All control cases had no evidence of HCC at the time the relevant serum sample was taken and within a minimum follow-up period of 12 months. Healthy volunteers were outpatients with normal liver biochemistry, no history of liver disease, and no malignant disease. Patients who received vitamin K antagonists were excluded from the study. Patients were excluded from the study if they had one or more of the following: 1) concurrent autoimmune disease, HIV or syphilis; 2) received vitamin K antagonists; 3) severe underlying cardiac or renal disease; or 4) clinical symptoms or signs of sepsis.

The tumor stages of HCC patients were defined according to the Union for International Cancer Control (UICC) TNM classification (8th version) 17. Among the 303 HCC patients, 156 were treated with hepatectomy, 70 were treated with transarterial chemoembolization (TACE), and 68 were treated with radiofrequency ablation (RFA).

### Serum assays and measurement

Blood samples were obtained from each participant at the hospital visit. For HCC patients, blood samples were withdrawn before treatment (D0), 7 days (D7) and 30 days after treatment (D30). All blood samples were centrifuged at 3000 rpm/minute for 10 min immediately after clotting and stored at -80 °C until analysis. The levels of AFP, AFP-L3, DCP and GP73 were measured via magnetic particle chemiluminescence immunoassay on an MQ60 Plus instrument (Beijing Hotgen Biotech, Beijing, China). The analytical sensitivity of the autoanalyzer is 0.6 ng/mL for AFP and AFP-L3. Serum AFP-L3 levels were expressed as the ratio of AFP-L3 to total AFP (%). Given that the lower limit of detection for AFP-L3 is 5%, nonmeasurable AFP-L3 was replaced by 5% for the analysis. The cut-off values used to establish positivity for AFP, AFP-L3, DCP and GP73 were 20 ng/ml, 5%, 40 ng/ml and 150 ng/ml, respectively, according to the manufacturer's instructions. The concentrations of interleukin 6 (IL-6) were analyzed by immunoassay using Human IL-6 Elecsys kits (Roche Diagnostics GmbH, Mannheim, Germany) according to the manufacturer's instructions and by an automatic biochemical immunoassay system (Roche Cobas 8000 e602). Serum amyloid A (SAA) was measured by fluorescent immunochromatographic assay (Weimi Bio-Tech, Guangzhou, China) according to the manufacturer's instructions.

### Follow-up

US and computed tomography (CT) were conducted one month after treatment and every 3-6 months thereafter. Extrahepatic organ examination was also carried out if patients had extrahepatic metastases. Liver magnetic resonance imaging was also used to define suspicious lesions demonstrated on CT. We defined recurrence as the appearance of new lesions with radiological features typical of HCC, as confirmed by at least two imaging methods for patients who underwent hepatectomy or RFA 18. For patients who underwent TACE, recurrence was defined as at least a 20% increase in the sum of the longest diameter of the target lesions or the appearance of new lesions or metastases.

### Statistical analysis

Data analyses were performed using SPSS 25.0 software (SPSS Inc., Chicago, IL, USA). Continuous data were presented as the mean ± standard derivation or median (interquartile ranges), and categorical data are expressed as frequencies. The Mann‒Whitney U test was used to compare quantitative variables, and differences between categorical variables were analyzed by chi-square tests or linear-by-linear associations. The Wilcoxon signed-rank test was used for paired samples. Multivariate logistic regression analysis was used to develop an index for predicting HCC. The receiver operating characteristic (ROC) curve was used to assess the diagnostic and prognostic performance of those serum tumor markers. Differences between the diagnostic and prognostic performance of serum tumor markers were compared with ROC curves and the area under the curve (AUC). The sensitivity, specificity, positive predictive value (PPV) and negative predictive value (NPV) were calculated to explore the best cut-off value. The best cut-off value was defined as the sum of the sensitivity and specificity that achieved its maximum. A *P* value ≤ 0.05 was considered significant.

## Results

### Patient characteristics

The characteristics of the study population are presented in Table 1. There was no significant difference in the distribution of age or sex among the four groups. A total of 90.8% of the patients in the HCC group and 93.2% and 83.7% of the patients in the HC group and LC group were infected with HBV respectively. In the HCC group, 133 (43.9%) patients were diagnosed with TNM stage I disease. Table S1 presents the associations between clinicopathological factors and the serum levels of the four biomarkers in HCC patients. All four biomarkers presented significantly higher serum levels in advanced-stage HCCs (TNM stage >I) than in early-stage HCCs (TNM stage I). Apart from DCP, AFP, AFP-L3 and GP73 had higher serum levels in poorly differentiated HCCs than in well-to-moderately differentiated HCCs.

### Ability of biomarkers in diagnosing HCC

**Figure 1:**
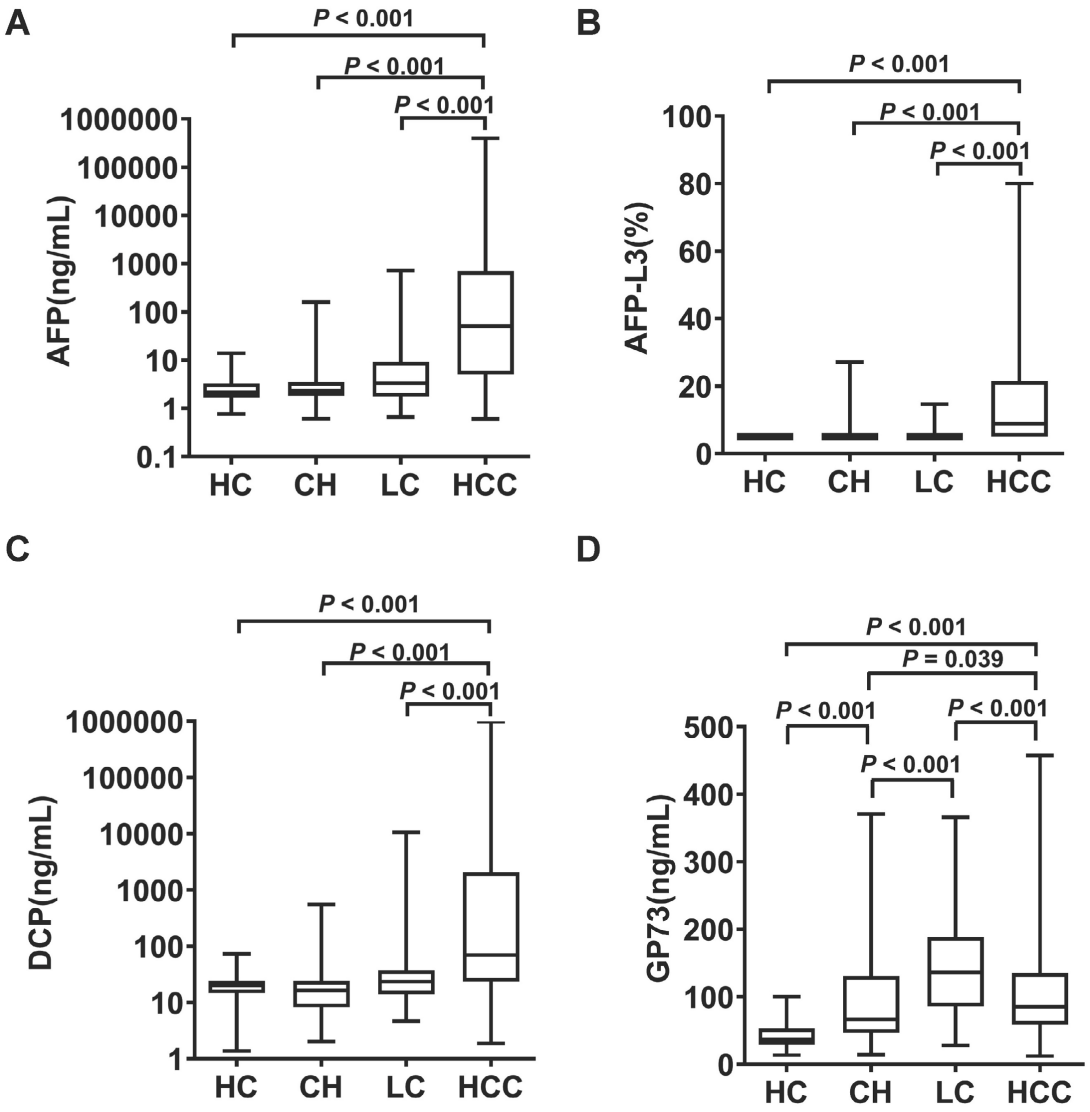
Serum levels of AFP **(A)**, AFP-L3 **(B)**, DCP **(C)** and GP73 **(D)** in healthy controls (HC) and in patients with chronic hepatitis (CH), liver cirrhosis (LC) and hepatocellular carcinoma (HCC). The box refers to the 25th and 75th percentile values, with a line indicating the median levels, while the whiskers extend from the box to show the range of the data.

**Figure 2:**
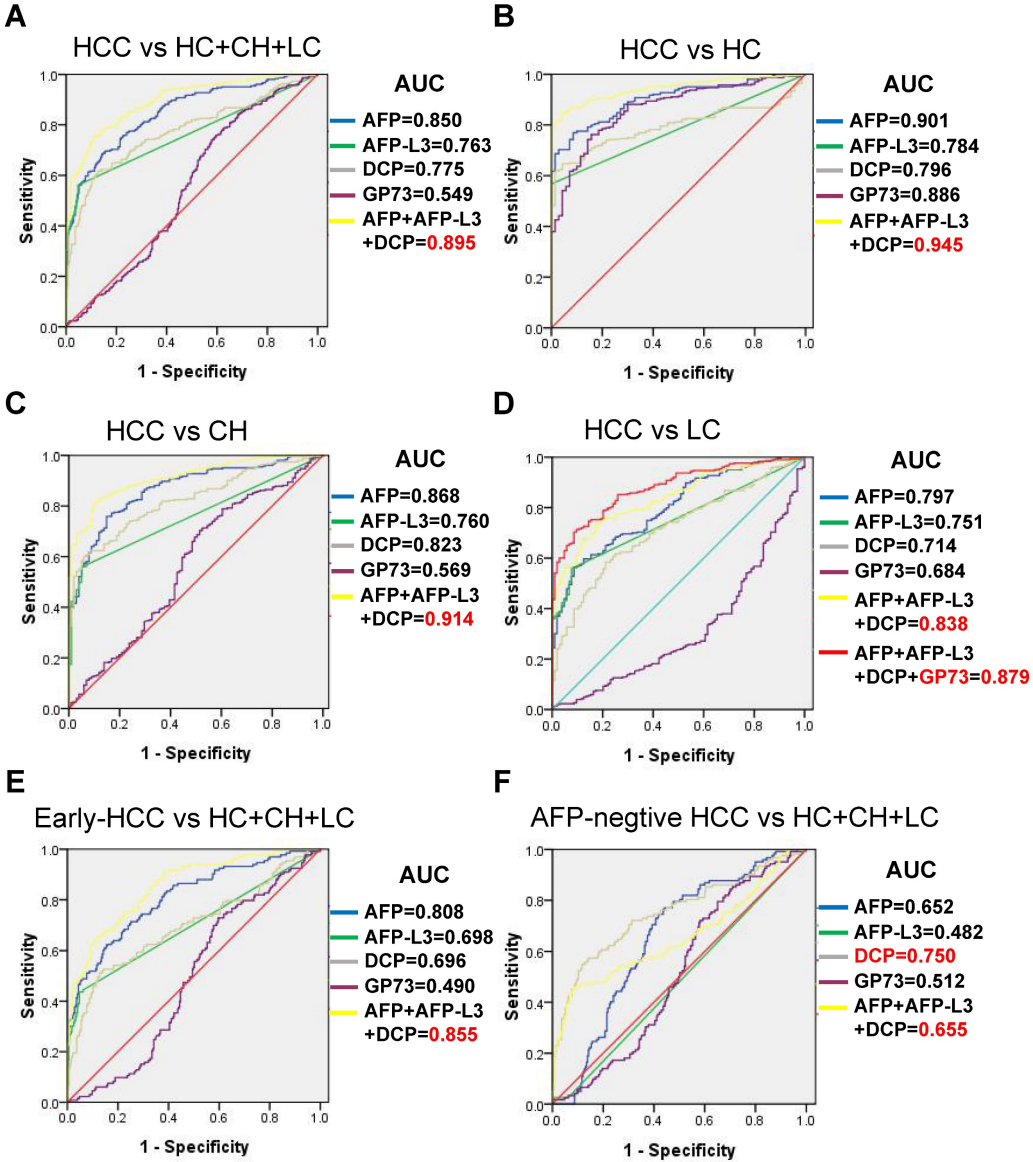
Comparison of receiver operating characteristic (ROC) curves of AFP, AFP-L3, DCP and GP73 for discrimination: **(A)** HCC vs HC+CH+LC; **(B)** HCC vs HC; **(C)** HCC vs CH; **(D)** HCC vs LC; **(E)** early-stage HCC vs HC+CH+LC; and **(F)** AFP-negative HCC vs HC+CH+LC.

The serum levels of AFP, AFP-L3, DCP and GP73 in different groups are summarized in Table 1. The serum levels of AFP, AFP-L3 and DCP were significantly higher in HCC patients than in control groups (Figure 1). In contrast, patients with LC had significantly higher GP73 levels than those with HCC. ROC curves were created to compare the diagnostic performance of the four biomarkers in detecting HCC. As shown in Figure 2A, AFP (AUC = 0.850, P < 0.001) was the most valuable predictor for discriminating HCC patients from all controls, followed by DCP (AUC = 0.775, P < 0.001) and AFP-L3 (AUC = 0.763, P < 0.001), whereas GP73 had low diagnostic efficiency (AUC = 0.549, P = 0.053). Diagnostic performance parameters, including sensitivity, specificity, PPV, and NPV, were calculated and presented in Table 2. The optimal cut-off values of the four serum markers were determined by maximizing the sum of sensitivity and specificity. The sensitivity of AFP (68.3%) was the highest among the single predictors, while the specificity of AFP-L3 (95.3%) was the highest. To further enhance the diagnostic performance, AFP, AFP-L3 and DCP were combined in a logistic regression model. The results implied that the prediction algorithm, which included AFP, AFP-L3 and DCP, had a greater AUC (0.895) than the individual biomarkers (Figure 2A).

We further examined the diagnostic value of the four biomarkers in discriminating HCC patients from different controls, and the results are shown in Figure 2B-2D. When the LC group was used as the control group, the diagnostic performance of AFP, AFP-L3 and DCP decreased slightly compared with that of the HC and CHB groups, but the diagnostic performance of GP73 was greater than that of the CHB group. Combining GP73 with AFP, AFP-L3 and DCP can improve the diagnostic performance in discriminating HCC versus LC, with an AUC of 0.879.

When only early-stage HCCs (TNM I) were evaluated, AFP had the best AUC (0.808), followed by AFP-L3 (0.698) and DCP (0.696), as shown in Figure 2E and Table 2. Moreover, we examined the differences in the diagnostic value of the four biomarkers in detecting AFP-negative patients with HCC (AFP < 20 ng/mL, n = 122), and the results are shown in Figure 2F. DCP achieved the best diagnostic performance in detecting AFP-negative HCCs, with an AUC of 0.750. In contrast, AFP-L3 presented no diagnostic value, whereas AFP alone and the combination of AFP, AFP-L3 and DCP presented similar low diagnostic values in detecting AFP-negative HCCs.

### Alterations in biomarkers after treatment and their ability to assess tumor recurrence

**Figure 3:**
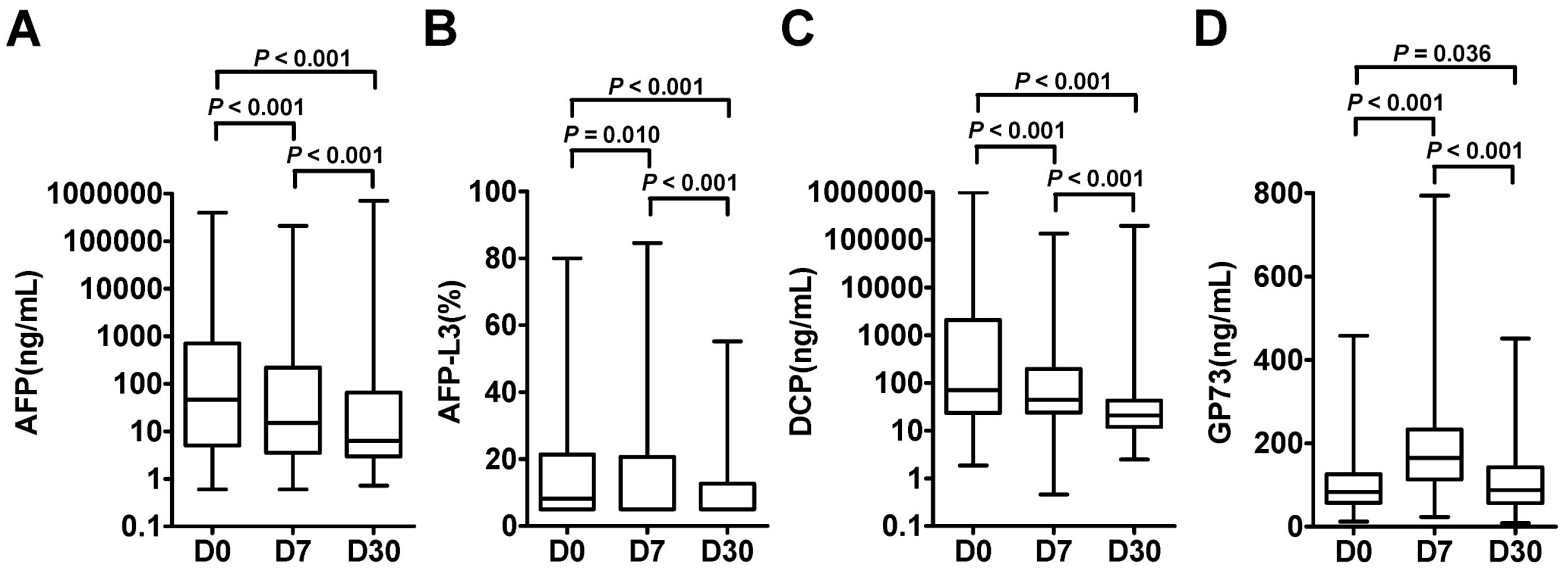
Dynamic changes of AFP **(A)**, AFP-L3 **(B)**, DCP **(C)** and GP73 **(D)** were evaluated in HCC patients on the days before treatment (D0), 7 days after treatment (D7) and 30 days after treatment (D30). The box refers to the 25th and 75th percentile values, with a line indicating the median levels, while the whiskers extend from the box to show the range of the data.

**Figure 4:**
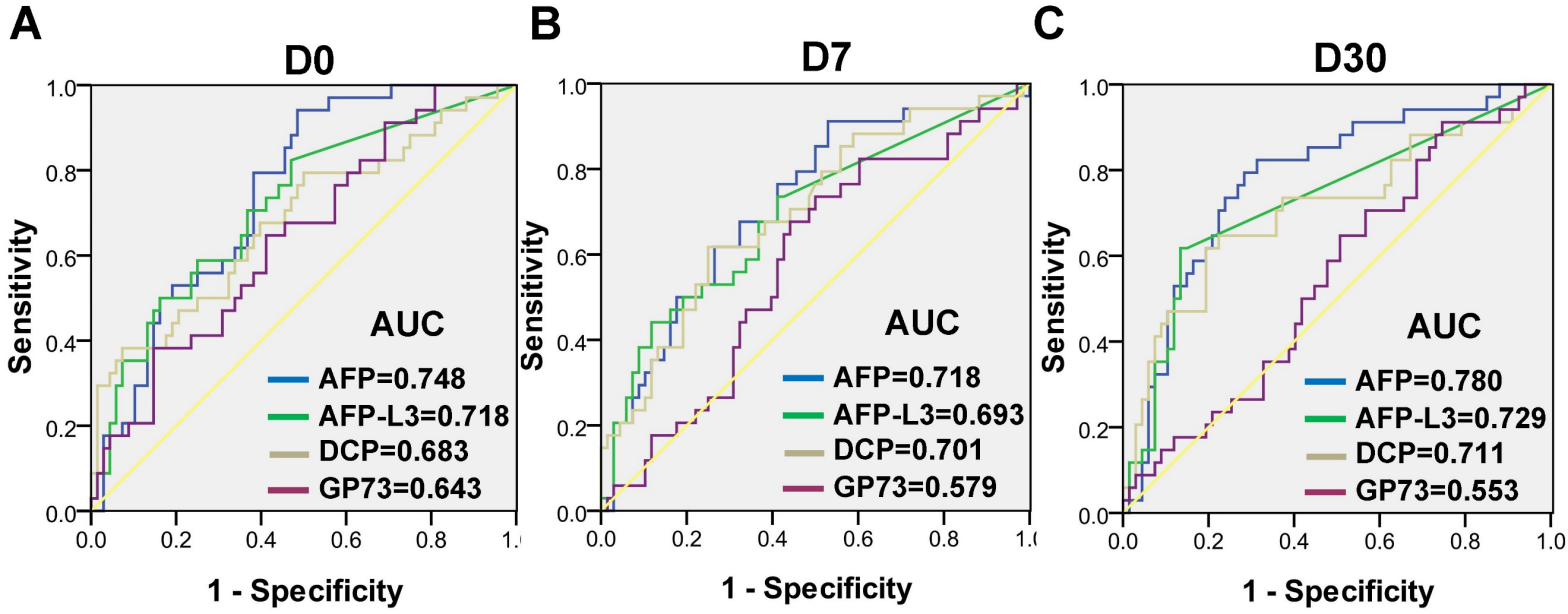
Receiver operating characteristic (ROC) curves of AFP, AFP-L3, DCP and GP73 before treatment **(A)**, 7 days after treatment **(B)** and 30 days after treatment **(C)** for assessing tumor recurrence.

The dynamic levels of the four biomarkers were evaluated in blood samples drawn from HCC patients the day before treatment (D0), 7 days after treatment (D7) and 30 days after treatment (D30) (Figure 3). The levels of AFP, AFP-L3 and DCP in HCC patients tended to decrease at D7 and D30, whereas the level of GP73 increased at D7 and then decreased at D30. Notably, the changes of inflammatory factors such as IL-6 and SAA were synchronized with those of GP73 (Figure S1). Among the 303 HCC patients, 102 patients with complete follow-up data and full-time point measurements of the four biomarkers were enrolled for assessing tumor recurrence. Among these patients, 68 remained free of recurrence one year after treatment. The rates of recurrence were compared according to the elevation of each tumor marker. The abilities of the four biomarkers to predict recurrence were depicted in Figure 4. AFP had the best predictive value (AUC = 0.748, 0.718 and 0.780, respectively) before, 7 days after and 30 days after treatment. We determined the rates of recurrence in patients on the basis of the number of elevated tumor markers before and after treatment. The cut-off points for each biomarker was set at the normal reference value. Higher recurrence rates were associated with an increasing number of elevated tumor markers measured both before and 30 days after treatment (P < 0.001), as determined by linear associations (Table 3). Furthermore, we analyzed whether the alterations of biomarker positive/negative status after treatment were related to the recurrence rate. The criteria used to determine the positive/negative status of these tumor markers were clarified in the methods section. As shown in Table 4, the recurrence rates of patients who still had marker-positive status 30 days after treatment were as follows: 20/33 (60.6%) for AFP, 20/30 (66.7%) for AFP-L3 and 15/21 (71.4%) for DCP. Whereas, the recurrence rates of patients whose positive marker status turned negative 30 days after treatment were as follows: 11/31 (35.5%), 8/30 (26.7%) and 12/42 (28.6%) for AFP, AFP-L3 and DCP, respectively. Thus, the risk of developing early recurrence in patients whose marker status remained positive 30 days after treatment was higher than that in patients whose marker status changed to negative according to the chi-square test, as shown in Table 4.

## Discussion

Biomarkers are key components of the clinical management of HCC patients, as they can contribute to early detection, major survival improvements and the optimization of medical interventions 19. Over the past years, AFP is the most commonly used biomarker for HCC surveillance. However, the specificity and sensitivity of AFP remain unsatisfactory, particularly for early HCC 20. Moreover, up to 40-50% of HCCs do not produce AFP, limiting the sensitivity of AFP alone for HCC detection. A large number of studies have identified other serum biomarkers that display promising diagnostic abilities to facilitate HCC detection and/or surveillance. The combination of biomarkers is recognized to increase the utility of individual biomarkers.

In this study, we investigated the diagnostic and prognostic value of the four serum biomarkers in HCC. Our results revealed that AFP was the most valuable predictor for both the diagnosis and the prediction of recurrence of HCC, followed by DCP and AFP-L3. Moreover, a prediction algorithm combining AFP, AFP-L3 and DCP presented enhanced diagnostic performance compared with individual biomarkers. Notably, the number of positive tumor markers and alterations in marker positive/negative status after treatment had good discriminatory ability for predicting tumor recurrence.

Consistent with many previous studies, we found that, compared with AFP-L3 and DCP, AFP was the most effective biomarker for the diagnosis of HCC, even early-stage HCC 10, 14, 21. However, the superiority of DCP over AFP has also been reported in other studies 22, 23. This difference may be due to the different backgrounds of the HCC patients and control groups in the different studies. Notably, AFP and DCP are independent markers and are thus thought to complement each other. In our study, we found that DCP still showed excellent diagnostic performance in detecting AFP-negative HCCs, whereas AFP and AFP-L3 presented no diagnostic value.

AFP-L3 is secreted by HCC cells even at early tumor stages and can be used in the absence of elevated AFP levels to detect early-stage HCC 24. Although high specificity has been reported as a feature of AFP-L3, AFP-L3 is limited by low sensitivity 25, 26. Our results showed that AFP-L3 had the lowest sensitivity (56.1%) and highest specificity (95.3%) in detecting HCC compared with AFP and DCP. When early-stage HCC (TNM I) was evaluated, AFP-L3 had an even lower diagnostic value than AFP. This may be due to the low sensitivity of the traditional AFP-L3 assay. These findings indicated that AFP-L3 may have limited utility as an independent diagnostic biomarker for HCC and must be combined with other biomarkers to increase the detection of early HCC. With the development of AFP-L3 detection technology, the sensitivity in the diagnosis of HCC may be improved in the future.

GP73 expression is upregulated in chronic liver diseases such as hepatitis, cirrhosis and HCC 27. Recent studies have identified serum GP73 as a promising biomarker for HCC 28. However, the ability of GP73 to discriminate between HCC and liver disease is controversial, as serum GP73 levels in liver cirrhosis patients decrease during HCC progression 29, 30. In the present study, we found the upregulation of serum GP73 in CH, LC and HCC groups compared to healthy controls. However, patients with LC had significantly higher GP73 concentrations than those with HCC, which may compromise its diagnostic accuracy. Studies have indicated that GP73 expression progressively rises throughout the progression of chronic liver disease. It is noteworthy that this increase occurs not only in hepatocytes but also in activated stellate cells which are the key cellular players in hepatic cirrhosis. This pattern explains why GP73 concentrations peak in the cirrhotic stage, exceeding those found in HCC 31, 32. GP73 can only improve the diagnostic performance in discriminating HCC versus LC. Whether the dynamic change of GP73 levels in patients with LC indicate the progression of HCC needs further study.

Typically, a good tumor marker should decrease to within a normal range after effective treatment and increase before tumor recurrence is detected by imaging. In the present study, the AFP, AFP-L3 and DCP levels in HCC patients decreased sharply after treatment, whereas the GP73 level increased immediately after treatment and then returned to the baseline level one month after treatment. The underlying mechanism of elevated GP73 may be related to inflammation after treatment. Interleukin 6 (IL-6) has been reported to be involved in this process. IL-6 promoted GP73 expression in HepG2 cells *in vitro* and promotes the transcription of GP73 and the preprotein convertase furin by binding to the IL-6 receptor to activate the JAK/STAT3 signaling pathway, and then the GP73 is freed from the Golgi membrane by cleavage 33. Consistently, we observed that the changes of IL-6 synchronized with those of GP73. Moreover, GP73 did not have significant predictive value for HCC progression. Taken together, these findings indicate that GP73 may not be a good tumor marker for assessing HCC recurrence.

The novelty of this study is that we evaluated the impact of dynamic changes of tumor markers on tumor recurrence before and after treatment. The serum level of AFP 30 days after treatment showed the best predictive value. All patients who were negative for the respective biomarkers before treatment also remained biomarker negative after treatment. In contrast, a large proportion of patients who were biomarker positive did not achieve marker negative status after treatment. A follow-up revealed that this was an unidentified sign of recurrence. A recent study reported that HCC patients who were positive for these three tumor markers before treatment have significantly lower recurrence-free and disease-specific survival rates after hepatectomy 34. However, post-treatment tumor marker levels were not assessed in this study. In the present study, we found that higher recurrence rates were associated with an increasing number of elevated tumor markers measured both before and 30 days after treatment. Notably, patients in whom all three tumor markers remained positive after treatment had the highest recurrence rates. These patients should be considered to have received ineffective treatment.

## Conclusion

AFP was shown to be a better biomarker than AFP-L3 and DCP both in HCC detection and predicting tumor recurrence. GP73 was not a good HCC marker under present clinical conditions. Combination of AFP, AFP-L3 and DCP enhanced the diagnostic performance. The dynamic changes of biomarkers positive status after treatment and the number of positive biomarkers play important roles in predicting HCC recurrence. Thus, we suggest that these three biomarkers should be assessed both before and after treatment. Nevertheless, the significance of combined detection warrants further research to be useful clinically.

## Supplementary Material

Supplementary figure and table.

## Figures and Tables

**Table 1:** Patient characteristics

Variables	HCC (n = 303)	HC (n = 70)	CH (n = 101)	LC (n = 104)
Age (years), mean±SD	54.2±11.7	55.1±12.2	53.4±11.2	55.2±10.7
Sex, males (%)	262 (86.5)	63 (90.0)	89 (88.1)	79 (76.0)
Etiology, n (%)				
HBV	275 (90.8)	0 (0)	96 (93.2)	87 (83.7)
HCV	10 (3.3)	0 (0)	4 (3.9)	5 (4.8)
other	18 (5.9)	0 (0)	1 (1.0)	12 (11.5)
ALT, median (IQR), U/L	33 (27)	17 (7)	34 (33)	31 (27)
AST, median (IQR), U/L	34 (27)	19.5 (6)	28.5 (22)	40.5 (40)
TBIL, median (IQR), μmol/L	12.4 (8.2)	9.6 (4.2)	10.1 (5.5)	22.0 (20.6)
ALB, median (IQR), g/L	39.8 (6.2)	46.8 (3.4)	45.6 (3.6)	36.7 (10.2)
ALP, median (IQR), U/L	79 (46)	65 (14)	84 (45)	96 (47)
PLT, median (IQR), 10^9^/L	168 (112)	236 (56)	176 (125)	81 (59)
Maximum tumor size, n (%)		NA	NA	NA
< 5 cm	122 (40.3)			
≥ 5 cm	181 (59.7)			
Tumor multiplicity, n (%)		NA	NA	NA
solitar	204 (67.3)			
multiple	99 (32.7)			
TNM tumor stage, n (%)		NA	NA	NA
I	133 (43.9)			
II	83 (27.4)			
III	33 (10.9)			
IV	54 (17.8)			
Vascular invasion, n (%)		NA	NA	NA
absent	221 (92.9)			
present	82 (27.1)			
AFP, median (IQR), ng/mL	50.5 (692.3)	2.2 (1.6)	2.3 (1.7)	3.3 (7.5)
AFP-L3, median (IQR), %	8.8 (16.5)	5.0 (0)	5.0 (0)	5.0 (0)
DCP, median (IQR), ng/mL	69.5 (2034.5)	20.0 (9.5)	16.3 (16.0)	23.4 (23.7)
GP73, median (IQR), ng/mL	85.3 (76.2)	37.1 (24.6)	66.5 (84.0)	136.1(102.9)

HCC, hepatocellular carcinoma; HC, healthy control; CH, chronic hepatitis; LC, liver cirrhosis; HBV, hepatitis B virus; HCV, hepatitis C virus; HBV, hepatitis B virus; HCV, hepatitis C virus; ALT, alanine aminotransferase; AST, aspartate aminotransferase; TBIL, total bilirubin; ALB, albumin; ALP, alkaline phosphatase; PLT, platelet; NA, not applicable; TNM, tumor-node-metastasis; AFP, alpha-fetoprotein; AFP-L3, lens culinaris agglutinin-reactive fraction of AFP; DCP, des-gamma-carboxy prothrombin; GP73, Golgi protein-73.

**Table 2:** Evaluation of the efficacy of serum AFP, AFP-L3 and DCP levels in the diagnosis of HCC

Parameter	All HCC						Early HCC					
	Cut-off value	AUC	SEN	SPE	PPV	NPV	Cut-off value	AUC	SEN	SPE	PPV	NPV
AFP	8.9	0.850	68.3	85.8	84.1	71.1	7.6	0.808	62.4	84.4	66.9	82.3
AFP-L3	6.3	0.763	56.1	95.3	92.9	66.4	6.3	0.698	43.6	94.9	80.5	77.7
DCP	36.9	0.775	61.7	87.3	84.2	67.4	35.5	0.696	52.6	85.8	63.7	78.9
AFP+AFP-L3 +DCP	-	0.895	74.9	89.5	88.7	76.4	-	0.855	65.4	88.0	72.5	84.0

AFP, alpha-fetoprotein; AFP-L3, lens culinaris agglutinin-reactive fraction of AFP; DCP, des-gamma-carboxy prothrombin; HCC, hepatocellular carcinoma; AUC, area under the curve; SEN, sensitivity; SPE, specificity; PPV, positive predictive value; NPV, negative predictive value.

**Table 3:** The relationship between the number of positive tumor markers and tumor recurrence before and after treatment

Pre-treatment			Post-treatment		
No. of positive tumor markers	No. of patients with recurrence (rate)	*p* value	No. of positive tumor markers	No. of patients with recurrence (rate)	*p* value
0	1/18(5.6%)		0	7/58(12.1%)	
1	5/25(20.0%)		1	12/21(57.1%)	
2	10/30(33.3%)		2	6/11(54.5%)	
3	18/29(62.1%)	P<0.001	3	9/12(75.0%)	P<0.001

**Table 4:** Alteration of marker positive/negative status through treatment and association with tumor recurrence

Pre-treatment		Post-treatment			
Marker status	No. patients (rate)	Marker status	No. patients (rate)	No. patients with tumor recurrence (rate)	*p* value
AFP (-)	38/102(37.3%)	AFP (-)	38/38 (100%)	3/38 (7.9%)	0.044
		AFP (+)	0/40 (0%)	0/0 (0%)	
AFP (+)	64/102(62.7%)	AFP (-)	31/64 (48.4%)	11/31 (35.5%)	
		AFP (+)	33/64 (51.6%)	20/33 (60.6%)	
					
AFP-L3 (-)	42/102(41.2%)	AFP-L3 (-)	42/42 (100%)	6/42 (14.3%)	0.002
		AFP-L3 (+)	0/0 (0%)	0/0 (0%)	
AFP-L3 (+)	60/102(58.8%)	AFP-L3 (-)	30/60 (50.0%)	8/30 (26.7%)	
		AFP-L3 (+)	30/60 (50.0%)	20/30 (66.7%)	
					
DCP (-)	39/102(38.2%)	DCP (-)	39/39 (100%)	7/39 (17.9%)	0.001
		DCP (+)	0/0 (0%)	0/0 (0%)	
DCP (+)	63/102(61.8%)	DCP (-)	42/63 (66.7%)	12/42 (28.6%)	
		DCP (+)	21/63 (33.3%)	15/21 (71.4%)	

Abbreviations: AFP, alpha-fetoprotein; AFP-L3, lens culinaris agglutinin-reactive fraction of AFP; DCP, des-gamma-carboxy prothrombin. *P*-value corresponds to patients whose marker status remained positive 30 days after treatment versus patients whose marker status changed to negative 30 days after treatment by chi-square.
