# Supplementary material for: Roles of AFP, AFP-L3, DCP and GP73 in Diagnosis of Hepatocellular Carcinoma and Prediction of Recurrence in Patients
Source: J Cancer. 2026 Jan 1;17(2):235–44. doi: 10.7150/jca.125861 (PMC12825132; doi:10.7150/jca.125861)
Supplement: Supplementary file 1 — Supplementary figure and table. [file jcav17p0235s1.pdf]

Figure S1 Dynamic changes of IL-6 (A) and SAA (B) were evaluated in HCC patients the day before treatment (D0), 7 days after treatment (D7) and 30 days after treatment (D30). The box refers to the 25th and 75th percentile values, with a line indicating the median levels, while the whiskers extend from the box to show the range of the data.

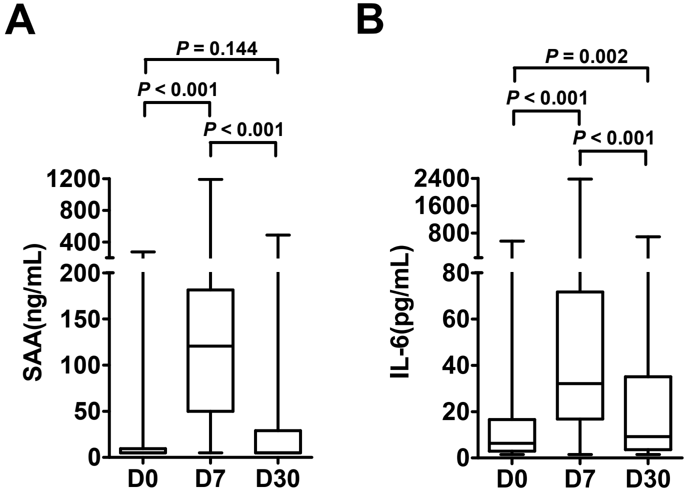

A
P = 0.144
P < 0.001
P < 0.001
1200
800
400
200
150
100
50
0
SAA(ng/mL)
D0
D7
D30
B
P = 0.002
P < 0.001
P < 0.001
2400
1600
800
80
60
40
20
0
IL-6(pg/mL)
D0
D7
D30

Table S1. Association between the serum levels of four biomarkers and clinicopathological factors in HCC patients

Variables	AFP		AFP-L3		DCP		GP73	
	Median (1st Qu. -3rd Qu.)	*p* value	Median (1st Qu. -3rd Qu.)	*p* value	Median (1st Qu. -3rd Qu.)	*p* value	Median (1st Qu. -3rd Qu.)	*p* value
Age(years)								
< 55	95.4(5.1-1125.7)	0.080	10.4(5.0-23.4)	0.089	47.2(20.5-1497.0)	0.054	74.7(51.3-114.6)	<0.001
≥ 55	30.2(4.7-467)		6.0(5.0-19.9)		125.8(26.3-2573.0)		93.7(69.0-147.3)	
Sex								
Male	42.3(5.0-661.7)	0.799	8.9(5.0-21.7)	0.425	71.3(24.6-2178.5)	0.228	85.7(58.4-133.8)	0.734
Female	73.6(4.5-904.6)		7.04(5.0-18.4)		40.13(18.1-871.0)		77.3(61.2-140.7)	
Tumor size								
< 5	31.1(4.6-306.3)	0.001	6.9(5.0-17.8)	0.003	28.6(17.2-115.8)	<0.001	75.5(50.3-105.6)	<0.001
≥ 5	150.8(5.3-3251.4)		12.4(5.0-31.6)		2240.0(176.7-11167.0)		104.9(71.2-170.5)	
Tumor number								
1	44.8(4.6-475.5)	0.037	7.14(5.0-20.5)	0.074	53.0(21.2-983.5)	0.001	79.2(56.8-164.6)	<0.001
≥ 2	62.6(6.6-2383.8)		12.0(5.0-22.5)		275.7(27.4-6129.8)		99.4(67.5-170.0)	
Tumor differentiation								
Poorly	278.5(12.1-6730.1)	0.026	24.0(5.0-32.1)	0.002	45.4(28.3-795.0)	0.758	102.8(63.6-170.6)	0.012

Well/moderately	42.1(4.1-334.7)		7.2(5.0-17.5)		57.4(23.6-861.6)		76.5(64.6-112.7)	
Vascular invasion								
Absent	30.8(4.6-381.1)	<0.001	6.10(5.0-18.3)	<0.001	46.2(20.5-510.3)	<0.001	82.5(57.3-120.9)	0.032
Present	185.0(12.8-236.5)		14.8(5.0-31.2)		916.7(39.1-10928.0)		93.4(64.7-154.3)	
Lymph node metastasis								
Absent	41.8(4.2-702.1)	0.038	7.19(5.0-20.2)	0.013	60.6(22.4-1808.8)	0.069	84.7(57.8-125.1)	0.140
Present	160.1(21.5-715.2)		13.5(5.3-27.4)		409.3(28.2-2439.0)		94.3(64.3-155.2)	
TNM tumor stage								
Stage I	20.3(3.5-291.9)	<0.001	5.0(5.0-15.0)	<0.001	37.0(16.9-327.7)	<0.001	76.6(54.6-110.6)	0.001
Stage II-IV	80.1(7.5-1628.5)		12.5(5.0-25.9)		341.6(28.3-6091.3)		88.9(63.4-153.9)	

AFP, alpha-fetoprotein; AFP-L3, lens culinaris agglutinin-reactive fraction of AFP; DCP, des-gamma-carboxy prothrombin; GP73, Golgi protein-73; TNM, tumor-node-metastasis.
